# Temporal dynamics in the free-living bacterial community composition in the coastal North Sea

**DOI:** 10.1111/1574-6941.12003

**Published:** 2012-09-17

**Authors:** Eva Sintes, Harry Witte, Karen Stodderegger, Paul Steiner, Gerhard J Herndl

**Affiliations:** 1 Department of Biological Oceanography, Royal Netherlands Institute for Sea ResearchDen Burg, The Netherlands; 2 Department of Marine Biology, Faculty Center of Ecology, University of ViennaVienna Austria

**Keywords:** annual cycle, community composition, environmental factors, free-living bacteria, T-RFLP

## Abstract

The coastal North Sea is characterized by strong seasonal dynamics in abiotic and biotic variables. Hence, pronounced temporal changes in the bacterioplankton community composition can be expected. Catalyzed reporter deposition fluorescence *in situ* hybridization analysis showed a seasonal succession, with Alphaproteobacteria dominating before the spring phytoplankton bloom, Bacteroidetes increasing during the bloom (up to 60% of the prokaryotic community) and being replaced by Gammaproteobacteria during the postbloom period (on average 30% of prokaryotic cells). Daily changes in similarity of the bacterioplankton community assessed by Terminal Restriction Fragment Length Polymorphism averaged 0.08 day^−1^ (Whittaker similarity index) for the free-living bacterial community, resulting in a decreasing similarity between samples with increasing time up to approximately 150 days. After about 150 days, the community composition became increasingly similar to the initial composition. Changes in the bacterial community showed periods of fairly stable composition, interrupted by periods of rapid changes. Taken together, our results support the notion of a recurring bacterioplankton community in the coastal North Sea and indicate a tight coupling between the resources, the bacterial community metabolism, physiological structure and community composition throughout the seasonal cycle in the coastal North Sea.

## Introduction

The regulation of microbial diversity and its relation to the functional stability of ecosystems is a pertinent question in ecology (Loreau *et al*., 2001; Hooper *et al*., 2005; Wittebolle *et al*., 2009). The temporal and spatial dynamics in the community composition of marine Bacteria and Archaea are governed either by physical factors (temperature and salinity), via substrate supply or by viral lysis and flagellate grazing (Fuhrman *et al*., 2006; Martiny *et al*., 2006; Alonso-Saez & Gasol, 2007; Alonso-Saez *et al*., 2008; Fuhrman & Steele, 2008). In systems where rapid changes in physical variables occur such as in estuaries, it has been shown that the biotic control of microbial diversity is of minor importance compared with the physical factors (Kirchman *et al*., 2005; Teira *et al*., 2008; Jackson & Vallaire, 2009). Also, variations in substrate supply might trigger changes in microbial diversity, although this aspect has not been studied extensively, except in mesocosm experiments.

The coastal North Sea is a eutrophic environment, characterized by distinct seasonal patterns in hydrography, inorganic nutrient concentrations and phytoplankton community composition and production (Brussaard *et al*., 1995; Cadée & Hegeman, 2002; Alderkamp *et al*., 2006; Sintes *et al*., 2010). This highly dynamic system is particularly suitable to investigate the strength of the link between prokaryotic community composition and environmental variables. Previous studies based on denaturing gradient gel electrophoresis (DGGE), ribosomal intergenic spacer analysis (RISA), pyro-sequencing or fluorescence *in situ* hybridization (FISH) have reported seasonal variations in the bacterial community composition in the North Sea (Eilers *et al*., 2000; Gerdts *et al*., 2004; Sapp *et al*., 2007; Gilbert *et al*., 2009). A study by Gerdts *et al*. (2004) on the bacterial community composition in the German Bight over a 3-year period identified three phases per year, a stable ‘winter’ period, a period with dramatic changes (between April and July) and a period where the changes seemed to slow down (August–September). The main factors reported to drive these changes in the bacterial community in the North Sea are abiotic variables, such as temperature and nutrients, and biotic such as phytoplankton (Sapp *et al*., 2007; Gilbert *et al*., 2009).

The objective of this study was to assess the bacterial community composition in the coastal North Sea with a sufficiently high temporal resolution to determine whether these changes in bacterial community composition occur gradually or whether periods of stable community composition are interrupted by abrupt changes. A previous study performed during a *Phaeocystis* bloom in the coastal North Sea pointed toward a stable bacterial community composition for a certain period of time followed by sudden changes (Arrieta & Herndl, 2002). However, the sampling interval used in the previous study did not resolve any temporal changes in the bacterial community composition (Arrieta & Herndl, 2002). In this study, we sampled coastal North Sea water up to twice per week to determine whether the variations in the bacterial community composition are gradually or abrupt and relate these changes to biotic and abiotic parameters over a seasonal cycle.

## Material and methods

Water samples were collected with an acid-rinsed bucket from the NIOZ jetty, located at the southern entrance of the North Sea into the Dutch Wadden Sea (53°00′18″N, 04°47′42″E), once per month and up to twice per week from December 18, 2002 to December 12, 2003 (Supporting Information, Table S1). Sampling was always carried out during high tide to collect incoming North Sea water.

Temperature and salinity were measured as part of the MARSDIEP monitoring series (Cadée & Hegeman, 2002) with a calibrated thermometer and a salinometer, respectively. Water samples used for biological and chemical analyses were prefiltered through a 55-μm Nitex screen to remove large particles including zooplankton. Inorganic nutrients, dissolved organic matter, phytoplankton composition and primary production, prokaryotic abundance, heterotrophic prokaryotic production, flagellate and viral abundance and viral production were measured as described elsewhere (Sintes *et al*., 2010). Briefly, the concentrations of dissolved inorganic nutrients (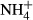, 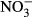, 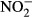 and 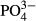) were determined after filtering the samples through 0.2-μm filters (Acrodisc, Gelman Science) in a TRAACS 800 autoanalyzer system. DOC, DON, and DOP concentrations were determined in triplicate GF/F filtered water samples using a Shimadzu TOC-5000 analyzer (Benner & Strom 1993, Valderrama 1981). Chlorophyll a (chl *a*) concentration was measured spectrophotometrically after filtering 0.5–1 L of prescreened water onto Whatman GF/F filters and extracting the pigments in acetone (90% v/v) in the dark at 4 °C for 24 h (Lorenzen 1967). Phytoplankton abundance and species composition were analyzed under a Zeiss inverted microscope on Lugol preserved samples (Philippart *et al*. 2007). Particulate phytoplankton production (PPP) and photosynthetic extracellular release (PER) were measured as described by Teira *et al*. (2001). Depth-integrated primary production was calculated as described by Philippart *et al*. (2007). Bacterial and flagellate abundance were determined by epifluorescence microscopy after DAPI-staining (Porter & Feig 1980), and viral abundance after SYBR Green I staining (Patel *et al*., 2007). To measure ^3^H-leucine incorporation by bacteria, subsequently referred to as heterotrophic prokaryotic production (HPP), two 5 mL samples and 1 formaldehyde-killed blank were inoculated with ^3^H-leucine (20 nM final concentration; specific activity 160 Ci mmol–1; Amersham) and incubated in the dark at *in situ* temperature for 4 h. Subsequently, the samples were fixed with formaldehyde (2% final concentration), filtered onto 0.45-μm Millipore HA filters, and rinsed three times with 10 mL of 5% ice-cold TCA. DPMs were used to calculate the molar incorporation rates of leucine that were subsequently converted to bacterial carbon production using the empirical conversion factor 0.07 × 10^18^ cells mol^−1^ Leu (Riemann *et al*. 1990) and assuming a bacterial C-content of 20 fg C cell^−1^ (Lee & Fuhrman 1987). Viral production was measured by the dilution approach (Wilhelm *et al*. 2002). Other methods used to analyze additional parameters are described briefly in Data S1. Based on the observed seasonal development of the biotic and abiotic variables, the seasons were defined as follows: prebloom period from December 18 to March 10, bloom period from March 18 to June 4, bloom decay period from June 10 to June 30, and postbloom period from August 4 to December 12 (Fig. S1).

### Bacterial community composition

#### CARD-FISH analysis

CARD-FISH analysis was performed as described elsewhere (Teira *et al*., 2004). Briefly, 5 mL seawater was fixed with 2% paraformaldehyde, kept overnight at 4 °C in the dark and filtered onto a 0.2-μm white polycarbonate filter (Millipore, Billerica, MA). The filters were dipped in low-gelling-point agarose [0.1% (w/v) in Milli-Q water], dried at 37 °C, and subsequently dehydrated in 95% (v/v) ethanol. For cell wall permeabilization, the filters were incubated in a buffer containing lysozyme (10 mg mL^−1^; Sigma, St. Louis, MO) at 37 °C for 1 h. Thereafter, filters were washed with Milli-Q water, incubated in 0.01 M HCl at room temperature for 20 min, rinsed with Milli-Q water, dehydrated with 95% ethanol, and air-dried. Filters were cut in sections for hybridization with HRP-linked oligonucleotide probes Eub338I-III (Bacteria), Non338 (negative control) (Amann *et al*., 1990; Daims *et al*., 1999), and the group-specific probes CF319 (Bacteroidetes) (Amann *et al*., 1995), ALF968 (Alphaproteobacteria), BET42a (Betaproteobacteria), and GAM42a (Gammaproteobacteria) (Manz *et al*., 1992). Hybridization was performed at 35 °C for 12–15 h and thereafter, the amplification at 37 °C for 30 min. Finally, filter sections were air-dried and stored at −20 °C until further processing.

### Bacterial DNA sampling and extraction

To sample selectively the free-living bacterial community, 2–5 L of seawater was prefiltered through a 3 μm polycarbonate filter (Millipore), concentrated to 50 mL by tangential flow ultrafiltration (0.2 μm PES, Vivaflow, Sartorius, Goettingen, Germany) and subsequently, pelletted by centrifugation (14 000 ***g*** at *in situ* temperature for 70 min). The pellet was resuspended in 2 mL of supernatant water. These cell concentrates were stored frozen (−80 °C) until DNA extraction was carried out. DNA from the cell concentrates was extracted with UltraClean Soil DNA Isolation Kit (MoBio, Carlsbad, CA). The centrifugation method was chosen vs. the normally used filtration method to collect bacteria as it has been shown to efficiently recover cells from environmental samples (Boström *et al*., 2004) and moreover, resulting in higher DNA extraction efficiencies than from samples collected on filters (Shimada *et al*., 2008; Janzon *et al*., 2009).

### Cloning, sequencing and phylogenetic analysis of bacterial 16S rRNA gene

The full-length bacterial 16S rRNA gene from one sample obtained during the bloom (April 7) and one sample from the postbloom (September 1) was amplified using the primers 27F (5′-AGA GTT TGA TCC TGG CTC AG-3′) and 1492R (5′-GGT TAC CTT GTT ACG ACT T-3′) (Lane, 1991). Thermocycling was performed as follows: initial denaturation at 94 °C for 4 min; amplification: 35 cycles, at 94 °C for 1 min, 55 °C for 1 min, and extension at 72 °C for 1 min, followed by a final extension step at 72 °C for 30 min and holding at 4 °C. The PCR product was purified using PCRExtract MiniKit (5-PRIME) and cloned with the TOPO-TA cloning kit® (Invitrogen) according to the manufacturer's instructions. Clones were checked for the right insert by running the PCR product on a 2% agarose gel. Sequencing was performed by MACROGEN Europe using the 27F and 1492R primers. Forward and reverse sequences were assembled together with CodonCode Aligner. The sequence data were compiled and aligned together with environmental bacterial sequences obtained from the NCBI database using Ribosomal Database Project (RDP, v10) online software (http://rdp.cme.msu.edu/). The phylogenetic affiliation of the bacterial 16S rRNA gene sequences was determined by Naïve Bayesian Classifier implemented in RDP (Wang *et al*., 2007), with a confidence threshold of 80%. The phylogenetic tree was built using the Neighbor-Joining method and Jukes-Cantor distance matrix in Mega-5 software and drawn with Interactive Tree Of Life (iTOL, v2.1) (Letunic & Bork, 2007). Rarefaction analysis and the Chao index were estimated using MOTHUR (Schloss & Handelsman, 2005; Schloss *et al*., 2009). Operational taxonomic units (OTUs) were defined as a group of sequences differing by < 3% as determined by the decrease redundancy software (http://www.expasy.org). Sequence information obtained in this study has been deposited in Genbank, accession numbers JX537799-JX537910.

### T-RFLP

Extracted DNA was used for bacterial community fingerprinting by T-RFLP (Moeseneder *et al*., 2001). The primers used for PCR were the bacteria-specific primer 27F-FAM and the universal primer 1492R-JOE (Lane, 1991). Each 50 μL PCR consisted of 0.25 μL of both primers, 5 μL of dNTP (Integra, Woburn, MA), 2% of DMSO, and 0.4 μL of Taq polymerase Biotherm D (Genecraft, Köln, Germany), 5 μL of the corresponding PCR buffer (Genecraft), and 1–2 μL of the DNA extract, made up to 50 μL with UV-treated ultrapure water (Sigma). Samples were amplified using an initial denaturation step at 94 °C (for 3 min), followed by 35 cycles of denaturation at 94 °C (1 min), annealing at 55 °C (for 1 min), and an extension at 72 °C (for 1 min). Cycling was completed by a final extension at 72 °C (for 30 min), followed by cooling at 4 °C (for 15 min) and maintaining the sample at 15 °C until further processing. The PCR products were purified on a 1.0% agarose gel after staining with SybrGold (Molecular Probes, Invitrogen, Carlsbad, CA). The PCR products in the expected size range (1500 bp) were excised from the gel and extracted with Qiaquick Gel Extraction kit (Qiagen, Hilden, Germany). FAM- and JOE-labeled PCR products were digested at 37 °C overnight. Each digest contained 20 ng of cleaned PCR product, 5U HhaI (Amersham Biosciences, GE Healthcare, Buckinghamshire, UK), and the recommended buffer (final reaction volume 50 μL). For T-RFLP analysis, the product of the restriction digest was desalted and subsequently denatured in the presence of 7.8 μL deionized formamide at 95 °C for 3 min. Additionally, each sample contained 0.2 μL Genetrace 1000 (ROX) marker (Perkin Elmer, Waltham, MA) for size determination of FAM- and JOE-labeled fragments.

Additionally, T-RFLP from clones containing plasmids with amplified 16S rRNA gene corresponding to distinct bacterial genera as determined from their sequences was carried on in the same way.

FAM- and JOE-labeled fragments were separated and detected with an ABI Prism 310 capillary sequencer (Applied Biosystems, Foster City, CA) run under GeneScan mode. Separation was performed in a noncoated capillary using POP4 polymer and sequencing buffer (Perkin Elmer). Injection was performed electrokinetically at 15 kV for 10 s, and the runs were completed at 15 kV within 45 min. Samples were detected with laser-induced fluorescence detection using the virtual filter set (A) of the 310 acquisition software.

Subsequently, the electropherograms were analyzed with Fingerprinting II software (Bio-Rad Laboratories, Hercules, CA). The threshold level to discriminate bands was set at 0.1% of the maximum peak height. The obtained matrix was analyzed by Primer software (Primer-E, Ltd, Ivybridge, UK) to determine the similarity between the different T-RFLP patterns obtained from the samples. Low-quality fingerprints were excluded from subsequent analysis, resulting in a total of 40 samples for the free-living bacterial community (Table S1).

### Diversity index

The Shannon-Wiener diversity index (*H′*) was calculated manually for each sample as:

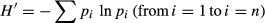

where *p_i_* is the relative contribution of the *i* OTU to the total DNA from one sample. Higher *H′* values indicate higher diversity.

### Statistics

Statistical analyses were performed with Primer 6.1.7 software (Primer-E, Ltd), Systat 12 (Systat Software Inc, Chicago, IL) and XLStat (AddinSoft SARL, Paris, France). Whole communities were compared by calculating the Jaccard, Bray–Curtis and Whittaker (*S*_w_) index of similarity. The first method considers the presence/absence of OTUs, while the two latter consider the relative contribution of each OTU to the total height. Jaccard and Bray–Curtis index of similarity were calculated using Primer software, and the Whittaker index was calculated manually using the equation given elsewhere (Legendre & Legendre, 1998):

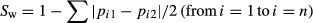

where *p*_*i*1_ and *p*_*i*2_ are the relative contributions of the *i* OTU to the total OTUs amplified DNA in samples 1 and 2, respectively.

The resulting matrixes were subjected to cluster analysis via the unweighted pair-group method using mean average (UPGMA) (Sokal & Rohlf, 1995). The Whittaker index was also calculated to assess the similarity between the community composition of adjacent dates. The mean daily change in similarity was calculated by first dividing the complement of similarity index (1-similarity index between two samples) by the separation in days between the two sampling dates, assuming linear change in similarity over the period between the two sampling dates.

One-way analysis of similarities was used to test for the significance of the seasonality of the community composition. Grouping of samples was carried out according to the previously described periods: prebloom, bloom, bloom decay, and postbloom.

### Analysis of patterns in resources, bacterial community metabolism, and structure

Four categories were defined for the analysis of the temporal patterns of the bacterial community in the coastal North Sea based on the categories described by Comte & del Giorgio (2009). These authors calculated dissimilarity matrices for different categories: a resource matrix, a community metabolism matrix, and four categories describing various aspects of the bacterial community structure, including bacterial community composition, metabolic capacity, based on substrate utilization profiles, bacterial physiological structure matrix, based on different single-cell activity indices, and a single-cell matrix based on flow cytometric characteristics of the bacterial cells (Comte & del Giorgio, 2009). MDS analysis was subsequently applied to these matrices to assess what components were ecosystem-specific or showed little or no distinct ecosystem patterns. The correlation between matrices was also used to assess the links between these categories. In this study, we analyze the links between four of the six matrices proposed by Comte & del Giorgio (2009). Each category was composed of a set of different variables (Table 1). The resources (Res), bacterial community metabolism (BCM) and bacterial physiological structure (BPS) distance matrices were generated from square root transformed data using Euclidean distance. The bacterial community composition (BCC) similarity matrix was generated from the relative fluorescence of T-RFLP fingerprints, normalized to the total fluorescence and square root transformed. Temporal patterns in resources and bacterial components were explored using MDS (Primer-E) on the basis of the calculated distance and similarity matrices of sampling dates running 500 permutations.

**Table 1:** Variables included in each of the distance or similarity matrices analyzed in this study

Matrix	Variables
Resource and environmental conditions (Res)	Dissolved organic carbon, Secchi depth, particulate and dissolved primary production, temperature, salinity, concentration of dissolved proteins, inorganic phosphorus, ammonium, nitrate, nitrite, dissolved organic nitrogen and phosphorus, chlorophyll *a*, diatoms and *Phaeocystis* cells
Bacterial community metabolism (BCM)	Rates of bacterial biomass production (Leucine incorporation), respiration, carbon demand, growth efficiency, D- and L-aspartic acid uptake, V_max_ and K_m_ from alpha-glucosidase, beta-glucosidase, aminopeptidase and phosphatase
Bacterial physiological structure (BPS)	Specific rates of leucine, D- and L-aspartic acid uptake and respiration, specific rates of alpha-glucosidase, beta-glucosidase, aminopeptidase and phosphatase, proportion of actively respiring cells (CTC+)
Bacterial community composition (BCC)	Relative contribution of each T-RFLP peak to the total relative fluorescence of the sample

### Links between resources, bacterial community metabolism and structure

Links between resources (Res), the bacterial community metabolism (BCM) and the two components of the bacterial community structure (BPS and BCC) were assessed by Mantel analysis comparing the corresponding distance/similarity matrices (Res, BCM and BPS: Euclidean distance matrices, BCC: Bray–Curtis similarity matrix).

### Relationships between community composition and environmental parameters

Pearson's correlation coefficients between all environmental and biological parameters were calculated for the whole dataset. The obtained *P* values were corrected for multiple testing using the Bonferroni correction (Ramette, 2007).

The T-RFLP distribution pattern obtained from the peak height of FAM- and JOE-labeled OTUs were normalized to the total fluorescence and subjected to principal component analysis (PCA). The first four axes of PCA explained 53% of the variation for the free-living communities obtained by T-RFLP. The sample scores obtained on these four axes were normalized to zero mean and standard deviation of 1, and most environmental parameters (except date, season, temperature, salinity, wind speed and direction, Secchi depth, and day-length) were log-transformed prior to the pairwise calculation of the Pearson's coefficients.

## Results

### Abundance of specific bacterial groups determined by CARD-FISH

**Fig 1:**
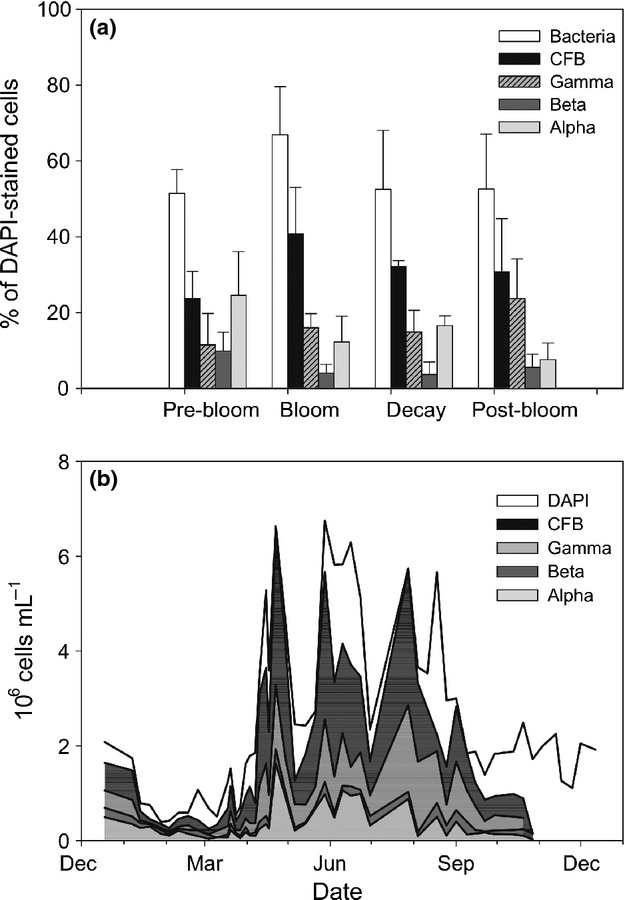
Mean percentage of Bacteria, Alpha-, Beta- and Gammaproteobacteria and Bacteroidetes (CFB) of DAPI-stained cells in the coastal North Sea for (a) the different seasons (prebloom, bloom, decay, and postbloom), and (b) abundance of the DAPI- stained cells (Sintes *et al*., 2010) and cumulative abundance of the different groups over the seasonal cycle. Lines in (a) indicate 1 SD.

Over the seasonal cycle, on average 58% of DAPI-stained cells (range: 35–84%) were identified as Bacteria. The percentage of Bacteria to DAPI-stained cells increased to 67% during the bloom (Fig 1a). Alphaproteobacteria varied between 1 and 40% of the DAPI-stained cells (annual average 14%) and were relatively more abundant during the winter (prebloom period), when their relative abundance was on average 25% of the DAPI-stained cells (Fig 1a and b). Betaproteobacteria varied from nondetectable to 20% of the DAPI-stained cells with an annual average of 6% (Fig. 1b). Similar to Alphaproteobacteria, Betaproteobacteria were also relatively more abundant during the prebloom (winter) period with an average contribution to DAPI-stained cells of 10% vs. 4–6% in the other periods (Fig. 1a). Alphaproteobacteria and Betaproteobacteria reached their maximum relative abundance in January (Fig 1b). Gammaproteobacteria exhibited similar relative abundances as Alphaproteobacteria, however, contrasting temporal dynamics, increasing during the postbloom period (Fig 1a and b). In terms of relative abundance, Bacteroidetes dominated the bacterial groups examined varying between 6 and 65% (annual average 33%) of DAPI-stained cells (Fig. 1b). Bacteroidetes was relatively more abundant during the spring bloom period (averaging 41% of DAPI-stained cells) than in the other seasons.

### Phylogenetic composition of the bacterial community

The Chao richness index was higher during the postbloom (205) than during the bloom period (125), although the contribution of major phylogenetic groups (Fig. S2) to the community composition during the two seasons was similar (Fig. S3). The 16S rRNA gene bacterial clone libraries from the bloom and the postbloom seasons were dominated by cyanobacteria and chloroplast-like sequences (Fig. S2) accounting for ~50% of the total sequences from the clone libraries. Alphaproteobacteria and Gammaproteobacteria were the more diverse and abundant bacterial groups followed by Bacteroidetes during both (bloom and postbloom) seasons (Fig. S2).

### Seasonal dynamics of the bacterial community composition

The temporal distribution of the different OTUs of the free-living bacterial community over the sampling dates is presented in Fig. S4 for FAM-labeled fragments obtained by T-RFLP. Four OTUs were always detected (56, 58, 91, and 514 bp), 18 OTUs appeared in more than 70% of the samples, and 10 OTUs only once (Fig. S4). Generally, the same trend in the distribution of OTUs was found for JOE-labeled fragments (data not shown). Some frequently present OTUs in the T-RFLP fingerprints were assigned to more than one bacterial genus based on the sequencing of clones. OTU 56 could be associated with one Gammaproteobacteria closely related to *Haliea*, and with several members of the family Rhodobacterales from Alphaproteobacteria (related to the genera *Roseovarius* and other unclassified genera). OTU 91 seemed to be related to microorganisms from different phyla, such as Deltaproteobacteria (related to *Bacteriovorax*), Bacteroidetes (*Fabibacter*), and Lentisphaerae (unclassified *Lentisphaeria*), and OTU 514 corresponded with cyanobacteria/chloroplast related to Chlorophyta, to members of the Bacteroidetes Sphingobacteriales family (*Balneola*, and another unclassified genera of Sphingobacteriales), to Gammaproteobacteria (two genera of *unclassified Gammaproteobacteria*) and to Alphaproteobacteria (*unclassified Alphaproteobacteria*). Other widespread OTUs were associated with the highly abundant cyanobacteria/chloroplast cluster (OTU 73, 1051), *Sulfitobacter* (OTU 369) and Gammaproteobacteria (*unclassified Gammaproteobacteria*, OTU 367).

Some OTUs were present only during specific periods of the seasonal cycle. For instance, the FAM-labeled OTUs 27 bp and 238 bp appeared throughout the seasonal cycle but not during the *Phaeocystis* bloom (Fig. S4). Other OTUs were present only during the bloom period such as OTU 227 bp (Fig. S4). Some Gammaproteobacteria (related to *Glaciecola* and *Methylophaga*) and Betaproteobacteria (*unclassified Comamonadaceae*), identified as OTU 202, did not appear during the postbloom period. Unfortunately, some OTUs could not be assigned to specific clones. Taken together, some OTUs were ubiquitously present, many others were present only during specific seasons, while others were not detected during specific periods such as the *Phaeocystis* bloom. Overall, there was a strong seasonality in the T-RFLP fingerprinting patterns (ANOSIM *r* = 0.51, *P* < 0.0001).

### Seasonal patterns in resources, bacterial metabolism and community structure

**Fig 2:**
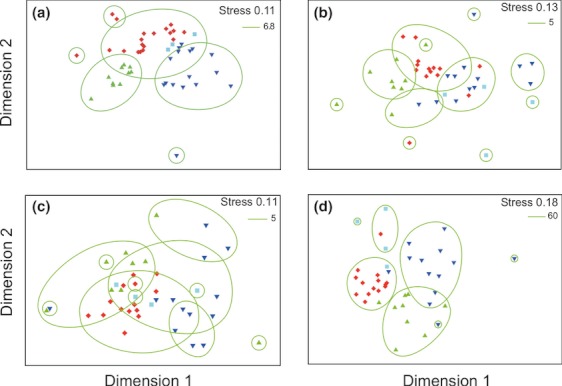
MDS ordination of dissimilarity (Euclidean distances) of samples collected at the different dates in terms of resources (a), bacterial community metabolism (b), bacterial physiological structure (c), and MDS ordination of similarity (Bray–Curtis) in terms of the free-living bacterial community composition (d). Symbols represent the different seasons: green triangles: prebloom, blue triangles: bloom, blue squares: bloom decay, red diamonds: postbloom. Lines engulfing samples from different dates represent the specified distance or similarity value.

Analysis of the resource matrix based on multidimensional scaling analysis (MDS) revealed a clear separation between the seasons in the coastal North Sea. Samples from the prebloom period clustered together and samples from the bloom period constituted a separate cluster, while samples from the postbloom, bloom decay period, and the end of the bloom period were located between these two clusters (Fig 2a), suggesting that the resource conditions during the postbloom period are intermediate conditions between prebloom and bloom. A similar pattern in the grouping of samples by seasons was obtained by MDS of the distance matrix of the bulk bacterial metabolism (Fig. 2b), and of the physiological structure of the bacterial community (Fig. 2c). The physiological structure of the bacterial community, however, was more overlapping between the different clusters. The MDS analysis of the similarity matrix obtained from the T-RFLP fingerprints revealed an even more distinct clustering of samples according to the seasons than that obtained based on the resources matrix. The prebloom, bloom, bloom decay, and postbloom bacterial communities were very well separated and arranged in a counterclockwise pattern (Fig. 2d).

### Temporal dynamics of bacterial communities

**Fig 3:**
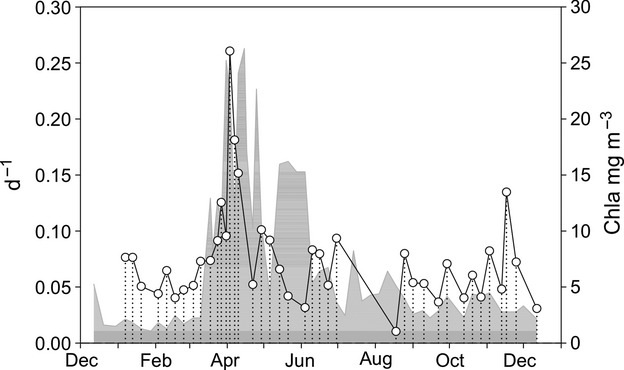
Mean daily changes in similarity of the composition of the free-living bacterial community collected at adjacent dates over the seasonal cycle. The average daily change is indicated by the dashed line, and chlorophyll *a* concentration is indicated by the shaded area.

The pairwise similarity (Whittaker index) between the bacterial assemblages from adjacent days changed during the seasonal cycle. The average daily change in similarity (Whittaker index) was 0.08 day^−1^ determined by T-RFLP. The daily change in similarity varied only over a fairly narrow range (between 0.01 and 0.13) over the seasonal cycle with a notable exception during the bloom period when changes of up to 0.26 day^−1^ were recorded (Fig. 3). The similarity between bacterial communities from contiguous sampling dates showed periods of slow (e.g. in the prebloom and postbloom period, Fig. 3) and rapid change (e.g. at the end of March, Fig. 3).

### Links between resources, metabolism and different components of the bacterial community structure

Mantel test comparison between all the different components of the bacterial community structure and the resources are presented in Table 2. All the components were significantly correlated with each other, with the exception of the bacterial physiological structure and the community composition. The strongest correlation was found between bacterial community composition and metabolism.

**Table 2:** Correlation coefficients (*R*) and significance level (*P*) obtained after Mantel test analysis between the distance matrices from resources (Res, Euclidean distance), bacterial community metabolism (BCM, Euclidean distance), bacterial physiological structure (BPS, Euclidean distance) and bacterial community composition (BCC, Bray–Curtis similarity)

	Res		BCM		BPS	
*R*	*P*	*R*	*P*	*R*	*P*
BCM	**0.226**	0.014				
BPS	**0.267**	0.006	**n.e**	n.e		
BCC	**0.283**	0.028	**0.354**	< 0.001	0.139	0.089

Significant correlations (*P* <** 0.05) are marked in bold. Correlations between BCM and BPS have not been evaluated (n.e.) as they might be autocorrelated (Table).

## Discussion

### Seasonal variations in the bacterial community

Enumeration of the main groups of Bacteria by CARD-FISH indicated shifts in their relative abundance associated with the spring phytoplankton bloom (Fig 1). Bacteria constituted ~60% of DAPI-stained cells (range: 35–85%) and thus contributed a similar fraction to the prokaryotic community as reported elsewhere for other coastal regions (e.g. Alonso & Pernthaler, 2005; Alonso-Saez *et al*., 2008). Generally, the bacterial contribution to the prokaryotic community was lower in winter, when the archaeal contribution is significant (Alderkamp *et al*., 2006; Wuchter *et al*., 2006) than in the summer. The seasonal dynamics in the abundance of the Bacteroidetes cluster were similar to those of the following year in the same area (Alderkamp *et al*., 2006) and in the German Bight (Pernthaler *et al*., 2001), with a significant increase in abundance during the *Phaeocystis* bloom, reaching a maximum of 63% of the bacterial community. This indicates a recurring temporal pattern in the seasonal variation in abundance of Bacteroidetes, and the adaptation of members of this group to utilize the high molecular weight DOM (Cottrell & Kirchman, 2000) released by *Phaeocystis* (Lancelot & Mathot, 1987; Mari *et al*., 2005).

The microbial community composition determined by T-RFLP also reveals a seasonal cycle with rapid changes associated with the *Phaeocystis* phytoplankton bloom (Fig 3). Specific clusters and OTUs, however, were found in all the seasons (Figs S2 and S3), similar to that reported for freshwater and marine systems applying both T-RFLP (Stepanauskas *et al*., 2003; Kritzberg *et al*., 2006; Anderson-Glenna *et al*., 2008) and ARISA (Kent *et al*., 2004; Fuhrman *et al*., 2006; Hewson *et al*., 2006; Fuhrman & Steele, 2008). Similarly to the four seasons differentiated in this study for the bacterial community composition, Gerdts *et al*. (2004) identified three phases over a year in the German Bight for bacterial communities characterized by DGGE fingerprinting, which re-occurred over a 3 years study period: a stable ‘winter’ period (from October to March) which would correspond to our pre- and postbloom periods, a period with dramatic changes (between April and July), corresponding to our bloom and bloom decay period, and a period where the changes seemed to slow down (August–September), corresponding to the beginning of our postbloom period.

Although a strong relationship between ARISA peak height and the abundance of *Prochlorococcus* has been reported (Brown *et al*., 2005), quantitative relationships between the relative peak height of specific prokaryotic OTUs do not necessarily reflect the actual contribution of these OTUs to the bacterial community. Blackwood *et al*. (2003) obtained a similar sensitivity in cluster analysis between fingerprints considering only the presence/absence of OTUs and with the peak height included. Thus, including peak height in T-RFLP fingerprints apparently does not necessarily lead to biases (Dunbar *et al*., 2000) in bacterial community composition analyses but rather accentuates trends (Sintes *et al*., unpublished) and should not have influenced the main findings reported in this study.

**Fig 4:**
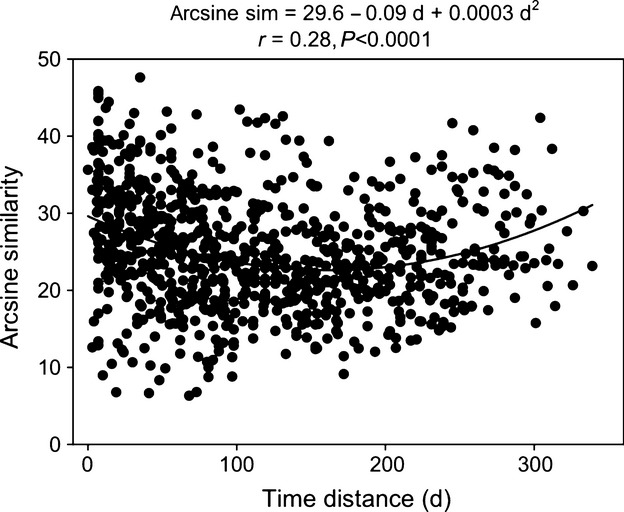
Pairwise similarity between free-living bacterioplankton community fingerprints vs. distance in time (in days, d).

The mean changes in the similarity indices of the bacterial community were 0.08 day^−1^ for T-RFLP and hence are lower than the 0.17 day^−1^ determined for open ocean surface oligotrophic waters (Hewson *et al*., 2006). More rapid changes (up to 0.26 day^−1^ for T-RFLP) were recorded during the initial phase of the phytoplankton bloom (Fig 3), accompanied by a decrease in the number of OTUs. Thus, the bacterial community composition exhibit periods of slow change and fairly sstable community composition, alternating with periods of rapid changes (Fig. 3). These rapid changes occur mostly during the onset of the Phaeocystis bloom (Sintes et al., 2010), coinciding with the increase in abundance of Cytophaga-Flavobacter (Fig. 3). These rapid changes occur mostly during the onset of the *Phaeocystis* bloom (Sintes *et al*.,) and a decrease in the number of OTUs (Sintes *et al*., unpublished). The daily changes in the community composition result in a significant decrease in similarity (*P* < 0.001) between the samples with increasing time separation (up to approximately 150 days) (Fig 4). Thereafter, the community composition becomes again more similar to the initial community, supporting the idea of recurring bacterial communities and their relationship with environmental factors (Fuhrman *et al*., 2006). Even though we have data for only 1 year, the presence of a recurring bacterial community composition is further supported by the MDS analysis (Fig 2d). The arrangement of samples from different seasons in a counterclockwise pattern on the MDS two-dimensional plot suggests a succession in the composition of the bacterial community from the prebloom toward the postbloom period, and returning to the community composition of the prebloom period (Fig. 2d).

### Environmental factors affecting the community composition in the coastal North Sea

Several statistical analyses can be used to determine the relationships between the patterns in the bacterial community composition and environmental parameters (Fuhrman *et al*., 2006). The principal component analysis (PCA) provides a definite value to the community composition of a specific sample, which can then be used to calculate correlations with environmental parameters (Ramette, 2007). These correlations provide a value and a positive or negative sign to the effect of the different environmental variables on the community composition (Table) and indicate the relation between different environmental parameters.

**Table 3:** Significant Pearson's correlation coefficient between environmental and biological parameters over the annual cycle at *P* < 0.05 after Bonferroni correction. SCORE1 and SCORE2 are the scores on the first two axes of the free-living bacterial community obtained by T-RFLP fingerprinting after principal component analysis

	Date	PO4	NO3	NH4	TN	Prot	PHAEO	BA_FL	BETA	VmaxBGase	VmaxLAPase	VmaxAPase
SCORE1	0.65	0.75		0.76			−0.69					
SCORE2			0.66		0.73	−0.63		−0.73	0.65	−0.69	−0.66	−0.66

PO4, inorganic phosphorus concentration; NO3, nitrate concentration; NH4, ammonium concentration; TN, total nitrogen; Prot, dissolved proteins concentration; PHAEO, total Phaeocystis cells; BA_FL, free-living bacterial abundance; BETA, abundance of Betaproteobacteria, VmaxBGase, VmaxLAPase; VmaxAPase, maximum enzymatic rate for Betaglucosidase, Leucine-aminopeptidase and Alkalino-Phosphatase, respectively.

The composition of the free-living bacterial community resulted in significant correlations with time (date and season), phosphate, ammonium, nitrate, total nitrogen, bacterial abundance and ectoenzymatic activity (Table 3). These results suggest that the community composition in the coastal North Sea is mostly governed by the available resources, in agreement with the bottom-up control of the bacterial metabolism and the inefficient control by predators in the coastal North Sea (Sintes *et al*., 2010). Our results further support previous findings showing that the changes of the bacterial community of the North Sea were caused not only by physical factors, such as temperature, but also by nutrients, especially phosphorus (Gilbert *et al*., 2009) and phytoplankton (Sapp *et al*., 2007).

Moreover, the resources, bacterial metabolism and the different components of the bacterial community structure were significantly related during the annual cycle in the coastal North Sea, suggesting that the bacterial metabolism, physiological structure and composition closely follow the seasonal variation of the resources in this environment throughout the year (Fig. 2 and Table 2). Previous studies have reported links between resources and metabolism or with the bacterial community structure (del Giorgio *et al*., 1997; Alonso-Saez *et al*., 2007; Bertilsson *et al*., 2007), but most of these studies focused only on particular components. Recently, Comte & del Giorgio (2009) found strong links between resources and metabolism, but not with bacterial community structure across a range of freshwater environments, suggesting a high degree of ecosystem specificity at the level of bacterial community composition. In contrast, in the coastal North Sea, the bacterial community composition as well as bacterial community metabolism and bacterial physiological structure all seem to be linked to the variations in the resources.

## Conclusion

The similarity (Whittaker index) between the samples changed at an average rate of 0.08 day^−1^, decreasing with time separation up to 150 day and increasing afterward again. This clearly supports the notion of an annually recurring bacterial community. Although the average daily change measured here was obtained from measurements on samples collected at intervals of several days, they allow distinguishing pronounced seasonal trends. Periods of slow and rapid changes in bacterial community composition were discernable. There is indication that changes in the bacterial community composition in the coastal North sea are linked to the variations in the resources, such as inorganic phosphorus, ammonium, nitrate and total nitrogen concentration, and bacterial abundance.

## Notes

Fig. S1 Seasons defined based on environmental and biological conditions.

Fig. S2 Phylogenetic tree based on 16S rRNA gene obtained from the bloom (green bars) and postbloom (red bars) periods in the coastal North Sea.

Fig. S3 Relative contribution from major bacterial phylogenetic groups to clone libraries from the bloom and postbloom periods in the coastal North Sea.

Fig. S4 Distribution pattern of OTUs indicated by filled squares determined by T-RFLP fingerprinting over the seasonal cycle in the coastal North Sea of the free-living bacterial community.

Table S1 Sampling dates and parameters measured in the coastal North Sea.
