# Supplementary figures and images for: Temporal dynamics in the free-living bacterial community composition in the coastal North Sea
Source: FEMS Microbiol Ecol. 2012 Sep 17;83(2):413–24. doi: 10.1111/1574-6941.12003 (PMC3561708; doi:10.1111/1574-6941.12003)

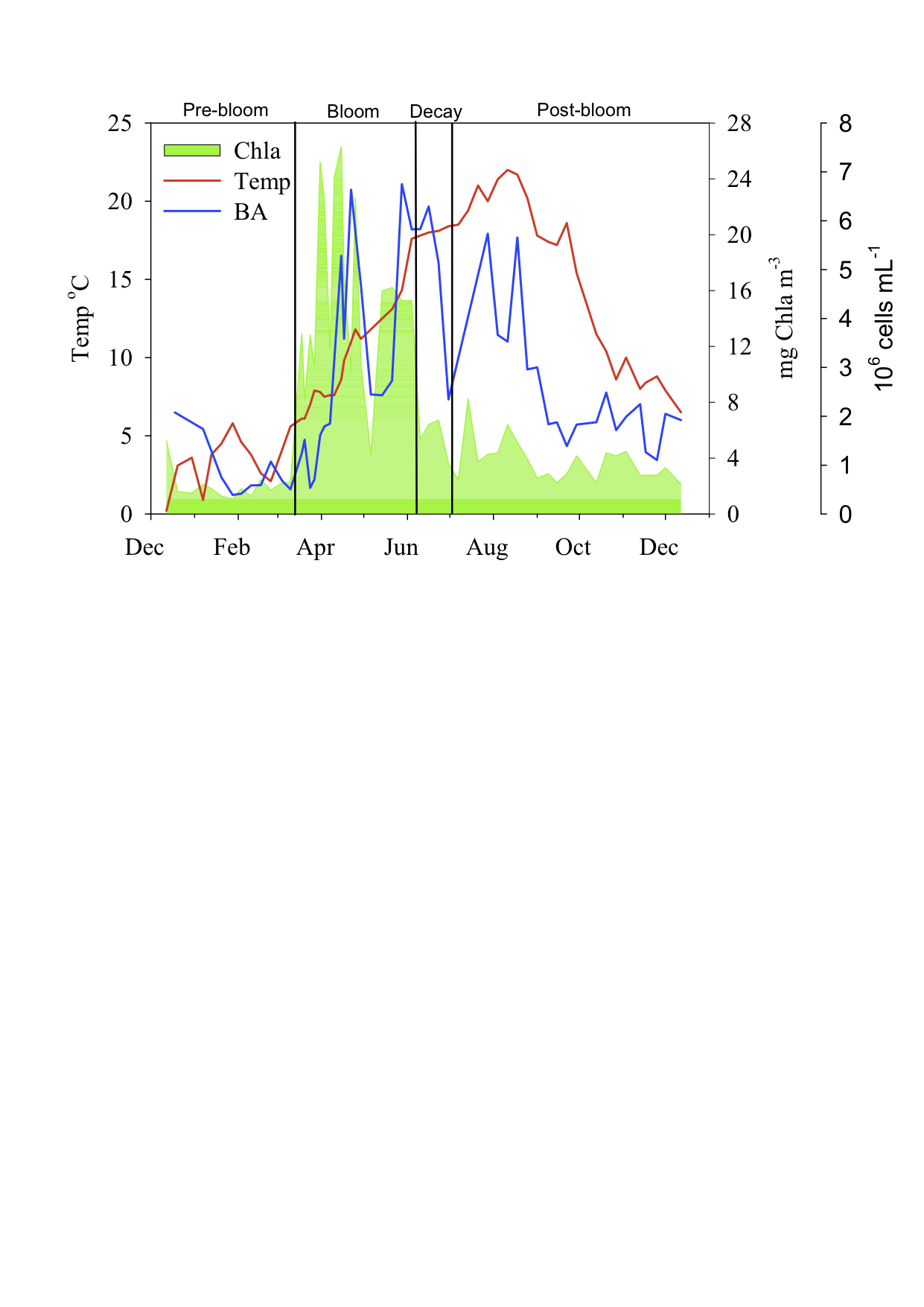

Supplement: Fig. S1 — Seasons defined based on environmental and biological conditions. [file fem0083-0413-sd1.tif]

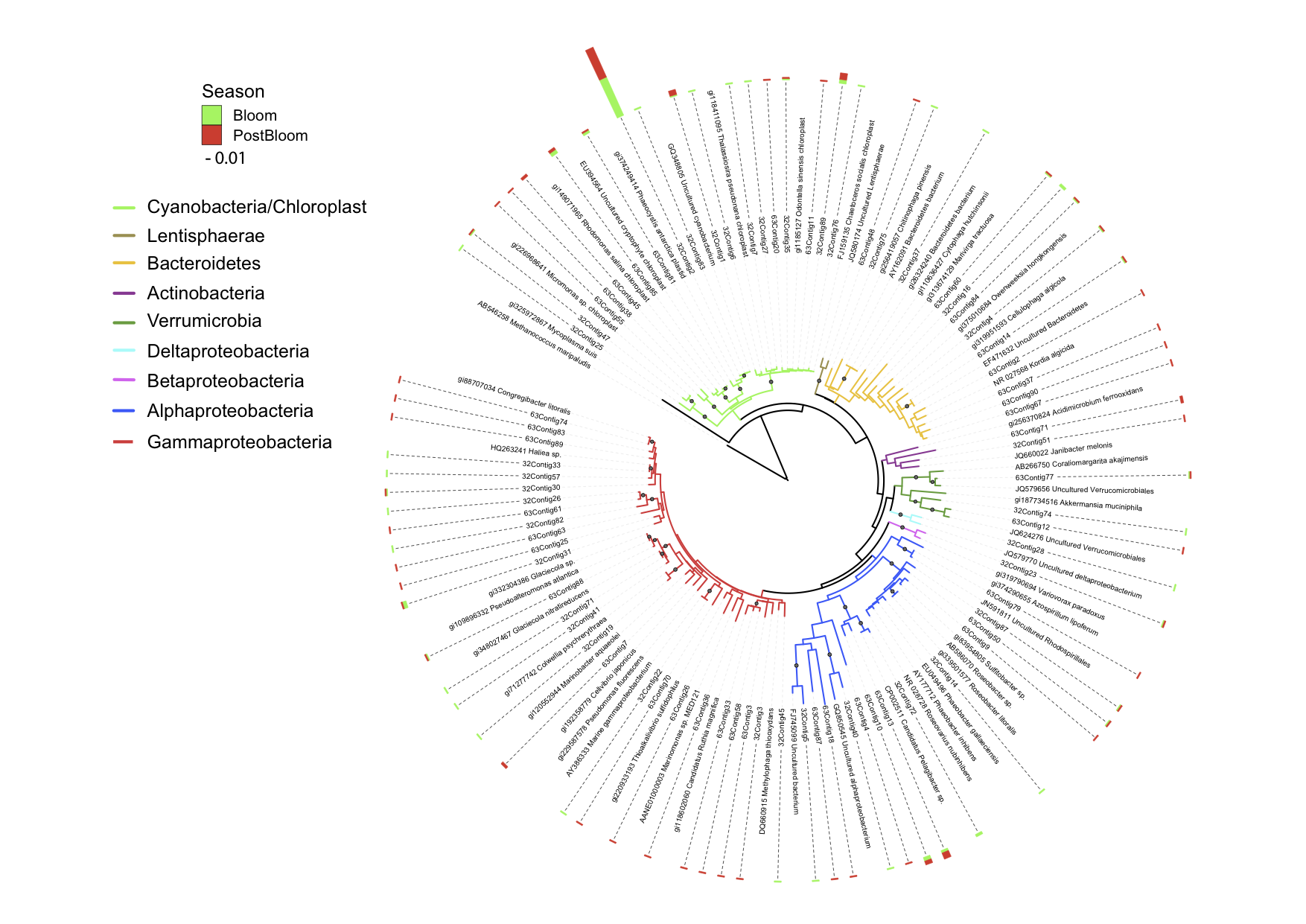

Supplement: Fig. S2 — Phylogenetic tree based on 16S rRNA gene obtained from the bloom (green bars) and postbloom (red bars) periods in the coastal North Sea. [file fem0083-0413-sd2.tif]

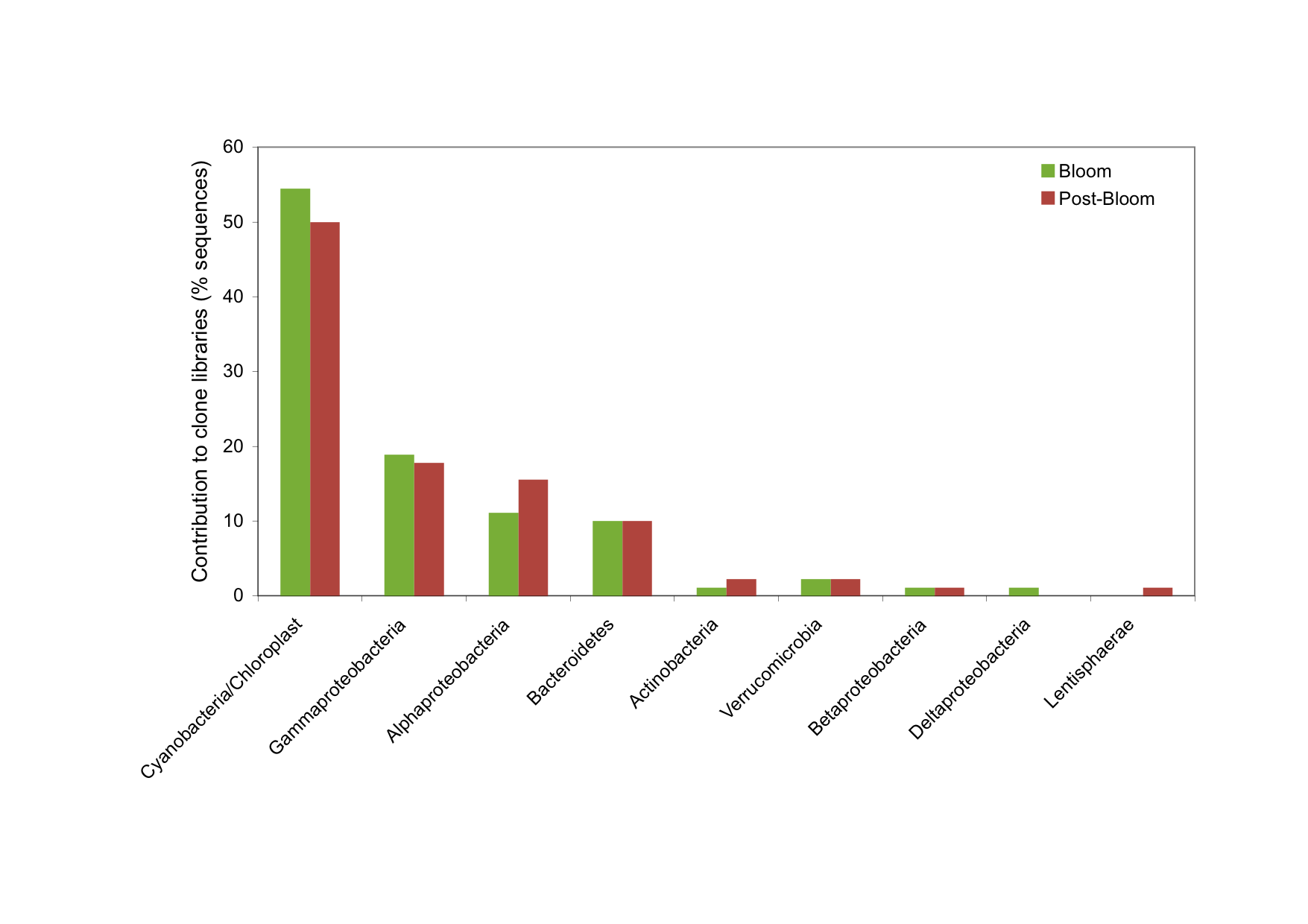

Supplement: Fig. S3 — Relative contribution from major bacterial phylogenetic groups to clone libraries from the bloom and postbloom periods in the coastal North Sea. [file fem0083-0413-sd3.tif]

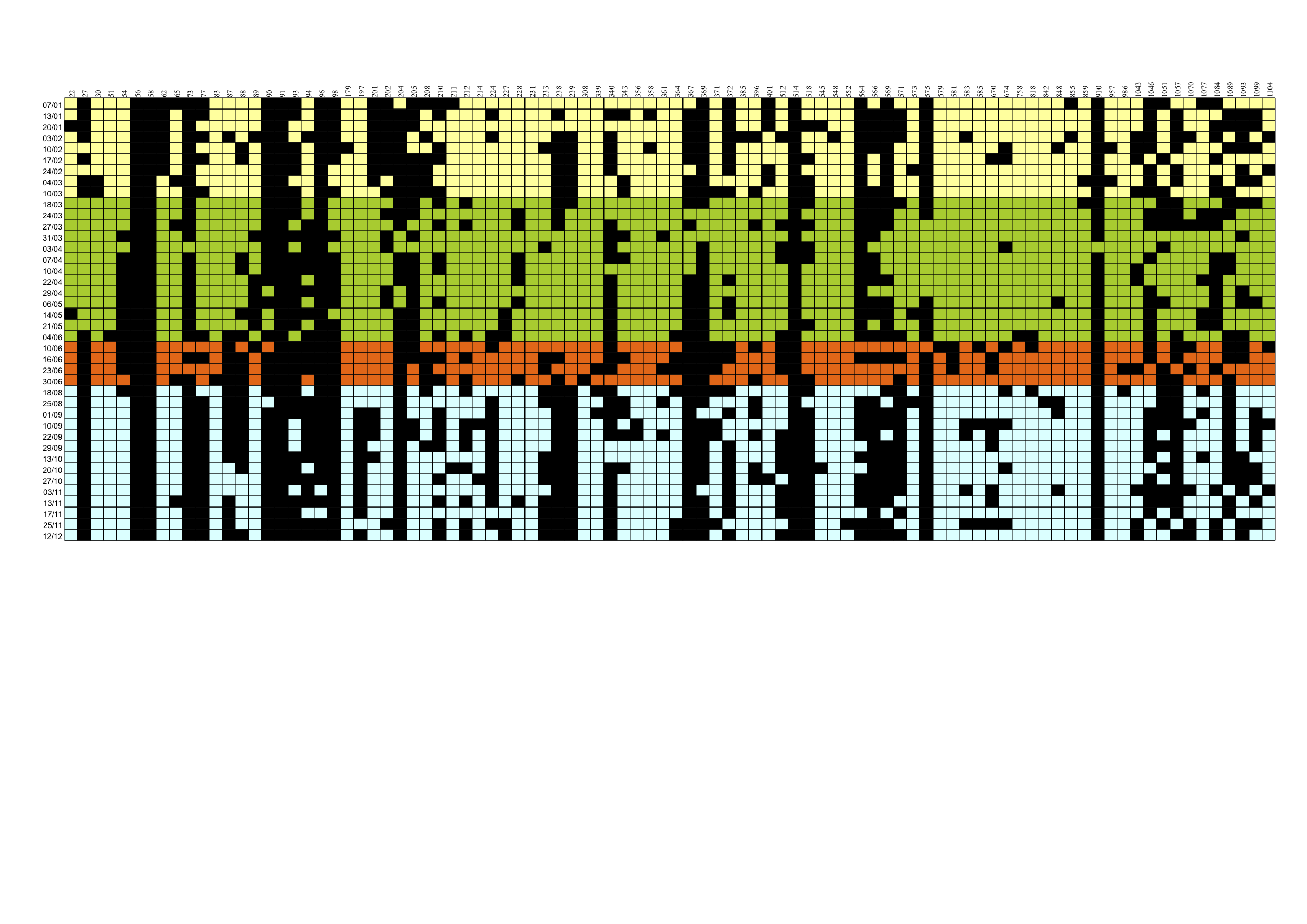

Supplement: Fig. S4 — Distribution pattern of OTUs indicated by filled squares determined by T-RFLP fingerprinting over the seasonal cycle in the coastal North Sea of the free-living bacterial community. [file fem0083-0413-sd4.tif]
