# Supplementary material for: Temporal dynamics in the free-living bacterial community composition in the coastal North Sea
Source: FEMS Microbiol Ecol. 2012 Sep 17;83(2):413–24. doi: 10.1111/1574-6941.12003 (PMC3561708; doi:10.1111/1574-6941.12003)
Supplement: Table S1 — Sampling dates and parameters measured in the coastal North Sea. [file fem0083-0413-sd5.doc]

Table S1. Sampling dates and parameters measured in the coastal North Sea. Seasonal dynamics of DOM and microbial activity was previously reported (Sintes et al., 2010). Additional unpublished parameters included in the analysis of patterns are indicated under other parameters. The dates when CARD-FISH and successful T-RFLP fingerprints were obtained are indicated.

| **Date** | **DOM and microbial activity** | **Other parameters** | **CARD-FISH** | **T-RFLP** |
| --- | --- | --- | --- | --- |
| 18 Dec | Temperature, salinity, inorganic nutrient concentration, chlorophyll *a*, phytoplankton abundance and primary production, DOC, DON, DOP, dissolved protein concentration, bacterial abundance, bacterial leucine incorporation, microbial respiration, flagellate abundance, viral abundance, viral production  (Sintes et al., 2010) | Secchi depth, wind speed and direction, day-length, D- and L-aspartic acid incorporation, Vmax and Km of α- and β-glucosidase, aminopeptidase and phosphatase, abundance of CTC+ cells  (Unpublished) | X |  |
| 1 Jan |  |  | X | X |
| 13 Jan |  |  | X | X |
| 20 Jan |  |  | X | X |
| 28 Jan |  |  | X |  |
| 3 Feb |  |  | X | X |
| 10 Feb |  |  | X | X |
| 17 Feb |  |  | X | X |
| 24 Feb |  |  | X | X |
| 4 Mar |  |  | X | X |
| 10 Mar |  |  | X | X |
| 18 Mar |  |  | X | X |
| 20 Mar |  |  | X |  |
| 24 Mar |  |  | X | X |
| 27 Mar |  |  | X | X |
| 31 Mar |  |  | X | X |
| 3 Apr |  |  | X | X |
| 7 Apr |  |  | X | X |
| 10 Apr |  |  | X | X |
| 15 Apr |  |  | X |  |
| 17 Apr |  |  | X |  |
| 22 Apr |  |  | X | X |
| 29 Apr |  |  | X | X |
| 6 May |  |  | X | X |
| 14 May |  |  | X | X |
| 21 May |  |  | X | X |
| 28 May |  |  | X |  |
| 4 Jun |  |  | X | X |
| 10 Jun |  |  | X | X |
| 16 Jun |  |  | X | X |
| 23 Jun |  |  | X | X |
| 30 Jun |  |  | X | X |
| 28 Jul |  |  | X |  |
| 4 Aug |  |  | X |  |
| 11 Aug |  |  | X |  |
| 18 Aug |  |  | X | X |
| 25 Aug |  |  | X | X |
| 1 Sep |  |  | X | X |
| 9 Sep |  |  | X | X |
| 15 Sep |  |  | X |  |
| 22 Sep |  |  | X | X |
| 29 Sep |  |  | X | X |
| 13 Oct |  |  | X | X |
| 20 Oct |  |  | X | X |
| 27 Oct |  |  | X | X |
| 3 Nov |  |  |  | X |
| 13 Nov |  |  |  | X |
| 17 Nov |  |  |  | X |
| 25 Nov |  |  |  | X |
| 1 Dec |  |  |  |  |
| 12 Dec |  |  |  | X |
